# Egr-1 regulates the transcription of NGX6 gene through a Sp1/Egr-1 overlapping site in the promoter

**DOI:** 10.1186/1471-2199-15-14

**Published:** 2014-07-16

**Authors:** Minji Liu, Xiaoyan Wang, Ya Peng, Shourong Shen, Guiyuan Li

**Affiliations:** 1 Department of Gastroenterology, Zhuzhou central Hospital, 412007 Zhuzhou, Hunan, China; 2 Department of Gastroenterology, Third Affiliated Hospital of Xiangya School of Medicine, Central South University, 410013 Changsha, Hunan, China; 3 Department of Digestive Diseases, Hunan Provincial People’s Hospital, 410005 Changsha, Hunan, China; 4 Cancer Research Institute, Central South University, 410078 Changsha, Hunan, China; 5 Hunan Key laboratory of Nonresolving Inflammation and Cancer, 410013 Changsha, Hunan, China

## Abstract

**Background:**

As a novel candidate metastasis suppressor gene, Nasopharyngeal carcinoma-associated gene 6 (NGX6) is involved in cellular growth, cell cycle progression and tumor angiogenesis. Previous studies have shown that NGX6 gene is down-regulated in colorectal cancer (CRC). However, little is known about its transcriptional regulation.

**Results:**

We defined the minimal promoter of NGX6 gene in a 186-bp region (from-86 to +100) through mutation construct methods and luciferase assays. Results from Electrophoretic mobility shift assays (EMSA) and Chromatin immunoprecipitation (ChIP) revealed that Early growth response gene 1 (Egr-1) binds to the Sp1/Egr-1 overlapping site of NGX6 minimal promoter. Overexpression of Egr-1 increased the promoter activity and mRNA level of NGX6 gene; while knock-down of endogenous Egr-1 decreased mRNA expression of NGX6 gene.

**Conclusion:**

These results demonstrate that Egr-1 regulates NGX6 gene transcription through an overlapping Sp1/Egr-1 binding site as a positive regulator of NGX6 gene.

## Background

Nasopharyngeal carcinoma associated gene 6 (NGX6) was located on chromosome 9p21-22 [1]. In previous studies, results from various analysis including RT-PCR, Dot hybridization and Northern blot showed that the mRNA levels of NGX6 were significantly lower in colorectal carcinoma tissues with lymph node or distant metastasis than that in paracancerous tissues [2]. And its mRNA expression level in nasopharyngeal carcinoma tissues was also lower than that in normal nasopharyngeal epithelial tissues [3]. Some studies demonstrated that NGX6 may play an important role in EGFR/K-ras/JNK/c-Jun/cyclinD1 signal pathway and Wnt/β-catenin signal pathway [4-6]. Overexpression of NGX6 gene in colon cancer cells was able to inhibit cell growth and cell cycle progression from G1 to S phase [7,8]. As a transmembrane protein, NGX6 protein has been demonstrated to regulate the transduction of extracellular signals into cytoplasm and nucleus through binding with the membrane cytoskeleton-organizing protein ezrin by its cytoplasmic domain. And it is also involved in cellular adhesion, invasion, motility and metastasis [9,10]. But its transcriptional regulation remains unknown. We have previously reported a region spanning from -159 to +276 bp as the proximal promoter of NGX6 gene [11]. In this report, we defined the minimal promoter of NGX6 gene in a 186-bp region, and explored the role of Egr-1 in positive regulating of NGX6 expression in colon cancer cells. These results will help to further understand and uncover the bio-functions of NGX6 gene involved in the pathogenesis of colorectal carcinoma.

## Methods

### Cell culture

The human colon carcinoma cell lines, HT-29, SW480 and SW620, were from American Type Culture Collection (ATCC, Rockville, MD). COS7 cells were provided by the Cancer Research Institute, Xiangya School of Medicine, Central South University (Human, P.R. China). All cells were cultured in RPMI1640 medium containing 10% heat-inactivated fetal bovine serum (FBS) and incubated at 37°C in a humidified incubator with 5% CO2.

### Bioinformatics

Potential binding sites of transcription factors within the promoter region spanning from -86 to +100 bp of NGX6 gene were analyzed by MatInspector Professional (http://www.genomatix.de).

### Luciferase- reporter vectors and assay

In order to define the minimal promoter of NGX6, a series of 5’ or 3’ deletion fragments generated from the proximal promoter construct pGL3-159/+276 were successfully amplified by PCR using the primers listed in Table 1. All the primers included 9-bp noncomplementary extensions capable of generating KpnI, HindIII or NheI restriction sites. These deletion fragments were cleaved, gel-purified and cloned into pGL3-enhancer vector (Promega). Five promoter plasmids (pGL3 -159/+100, pGL3 + 100/+276, pGL3 -159/-86, pGL3 -86/+12 and pGL3 + 12/+100) were verified by restriction enzyme cutting and sequencing.

**Table 1:** Primers used for generating NGX6 promoter constructs

pGL3-159/+100	Forward:5′- AAAGGTACCTGTGCTTGGGGTGAGAAA-3'
	Reverse: 5′- AAAGCTAGCGGGACCTGGGTAGGAGTT-3'
pGL3 + 100/+276	Forward: 5′- AAAGGTACCGCCTGAACTCCTACCCA-3'
	Reverse: 5′- AAAGCTAGCGGATTGGGATAGGACGAG-3'
pGL3-159/-86	Forward: 5′- AAAGGTACCTGTGCTTGGGGTGAGAAA-3'
	Reverse: 5′- AAAAAGCTTTTAGTCCTGCTGGGCTTC-3'
pGL3-86/+12	Forward: 5′- AAAGGTACCTCCTCGAAGCCCAGCAG -3'
	Reverse: 5′- AAAAAGCTTACTTGACGTCGGCGTGAC-3'
pGL3 + 12/+100	Forward: 5′- AAAGGTACCGCCGACGTCAAGTCGAG-3'
	Reverse: 5′-AAAAAGCTTGGGACCTGGGTAGGAGTTC-3'

Cells were seeded at 5 × 10^5^ cells/well and cultured in 12-well plates for 24 h prior to transfection. The cells were transfected with 1 μg of various NGX6 promoter constructs, pGL3-control plasmid, or pGL3-enhancer plasmid by Lipofectamine 2000 reagent (Invitrogen) according to manufacturer’s instructions. To control transfection efficiency, cells were co-transfected with 0.5 μg SV40 β-galactosidase vector per well. 48 h after transfection, the cell lysates were prepared and luciferase activity was measured by luciferase assay kit (Promega). β-galactosidase activity was also quantified using the β-galactosidase Enzyme Assay System (Promega). Experiments were repeated at least three times with three replicates per sample.

### Electrophoretic mobility shift assay (EMSA)

Nuclear protein was prepared by using NR-PER nuclear and cytoplasmic extraction reagents (Pierce Biotechnology, Rockville, IL). Then nuclear supernatants were collected and stored at -80°C until used. Protein concentration was determined using BCA protein assay kit (Pierce Biotechnology, Rochville, IL). And the following oligonucleotides and their complementary strands were synthesized and polyacrylamide gel electrophoresis (PAGE)-purified by Songan: NGX6 (-54/-39) 5′-GTAGG*GCGGGGGCG*GGCTTTACT-3′ (in which the G/C-rich sequences shown in italics are potential binding sites for the transcription factors for Egr-1 and Sp1).

According to the manufacturer’s instructions of lightshift chemiluminescent EMSA Kit (Pierce), 10 ug nuclear protein extracts were incubated with 50 fmol of biotin labeled oligonucleotides for 20 min at room temperature (20°C ~ 25°C). In competition experiments, prior to the addition of the labeled oligonucleotides nuclear extracts were incubated for 10 min with excess unlabeled oligonucleotides. The reaction mixtures were then resolved on 6% polyacrylamide gel in 0.5 × TBE. Then the binding reactions were transferred to nylon membrane and crosslinking was performed with a hand-held UV lamp equipped with 254 nm bulbs. Finally, the biotin-DNA was detected by chemiluminescence.

### Chromatin immunoprecipitation (ChIP) assay

ChIP assays were performed by using a kit from Upstate Biotechnology. HT-29 cells were crosslinked by ‘ing 1% formaldehyde in cell culture medium for 10 min at room temperature, followed by adding glycine to end the process. The cross-linked chromatin was sonicated to yield fragments of 200 to 1000 bp. Soluble chromatin was then clarified by centrifugation for 10 min at 14000 rpm at 4°C, and 1% of the supernatant was saved as input. Diluted soluble chromatin fragments were precleared with protein G agarose to discard nonspecifically bound chromatin fragments, then immunoprecipitated with antibodies against Sp1(07–645) (Upstate Biotechnology) or Egr-1(588) (Santa Cruz Biotechnology) overnight at 4°C. Immunocomplexes were captured on the ssDNA/protein G-agarose slurry, and washed sequentially with low-salt wash buffer, high-salt wash buffer and LiCI wash buffer, followed by two final washes with TE buffer. After washing, the immunocomplexes were eluted by incubation for 15 min at 25°C with 200 μl of elution buffer, and reversed for 6 h at 65°C. The DNA fragments were extracted with phenol/chloroform and precipitated with ethanol. The immunoprecipitated DNA samples were analyzed by PCR using the primer pair: (forward) 5′-AAAGGTACCTGTGCTTGGGGTGAGAAA-3′ and (reverse) 5′-AAAGCTAGCGGGACCTGGGTAGGAGTT-3′. PCR was carried out for 35 cycles by using a step cycle of 95°C for 30 sec, 55°C for 50 sec, 72°C for 1 min, and followed by 72°C for 10 min. A 259 bp product was detected from the reaction. As a negative control for PCR, water was added instead of the immunoprecipitated chromatin fragments. The PCR products were analyzed by electrophoresis on a 5% agarose gal.

### Construction of pCMV-HA/Egr-1

To construct wild type Egr-1 expression vector, Egr-1 gene cDNA was amplified by PCR method using human fetal brain cDNA library as template. According to the sequence of Egr-1 gene (NM_001964) obtained from GeneBank, the following primers with EcoRI and XhoI restriction enzyme sites were used: Egr-1 (forward) 5′-TTT*GAATTC*AGGATGGCCGCGGCCAAG-3′ and (reverse) 5′-CAC*CTCGAG*TTAGCAAATTTCAATTGTC-3′ (restriction sites underlined). After an initial denaturation step at 94°C for 10 min, the PCR was carried out for 38 cycles at 94°C for 30 sec, 58°C for 50 sec, 72°C for 2 min, and followed by 72°C for 10 min. The PCR fragments were purified and subcloned into EcoRI/XhoI-digested pCMV-HA vector, then transformed into competent JM109 cells. Positive clones were sequenced to verify the correct inserts, and then named as pCMV-HA/Egr-1.

### siRNA target to Egr-1

Egr-1 oligo small interfering RNA (siRNA) (5'-CCAUGGACAACUACCCUAA[dT][dT]-3') and a negative control siRNA (5'-ACUUACGAGUGACAGUAGA[dT][dT]-3') were purchased from GenePharma. For siRNA transfection, SW480 cells were plated overnight in 6-well dish at a density of 1 × 10^6^ cells/per well. They were transiently transfected with 100 nM siRNA using lipofectamine 2000 transfection reagent as described by the manufacturer (Invitrogen). The cell lysates were harvested 2 days after transfection. The total RNA from SW480 cells was isolated with the RNeasy Mini Kit (Qiagen, Hilden, Germany). Then Egr-1 expression and NGX6 expression were evaluated by RT–PCR or Real-time quantitative PCR.

### RT-PCR and Real-time quantitative PCR

Total RNA from harvested cells was isolated using Trizol reagent (Invitrogen) and then treated with DNaseI (Roche) to eliminate possible DNA contamination. RNA was quantified by optical density (A260) and stored at -80°C until used. cDNA was prepared using Reverse Transcription System (Promega).

2 μl single-stranded cDNA was amplified by PCR using NGX6-specific primers primers and glyceraldehide-3-phosphate dehydrogenase (GAPDH) primers. The GAPDH primers were added to the PCR at the end of the tenth cycle as control experiments. RT-PCR products were analyzed by electrophoresis on a 5% agarose gal.

Real-time quantitative PCR was performed using SYBR^®^ Premix Ex Taq™ (TaKaRa, Dalian, China) according to manufacturer’s recommendations. 96 wells plates were used on BIO-RAD IQ™5 thermocycler. Cycling conditions were as follows: an initial step at 95°C 5 min for enzyme activation, followed by 40 cycles alternation of 10 sec at 95°C, 15 sec at 54°C, 30 sec at 72°C and a final dissociation step. Obtained Ct values were normalized against GAPDH. Relative gene-expression was determined by using the △Ct method [12]. The primers were used in RT-PCR and Real-time qPCR were as follows: NGX6 forward (5'-AGAACCGCCATCCCTT-3'), NGX6 reverse (5'-CACCTCGTGAGTCAAGCA-3'), Egr-1 forward (5′-CACGAACGCCCTTACGCT-3'), Egr-1 reverse (5′-CATCGCTCCTGGCAAACT-3'), GAPDH forward (5'-AGGTCGGAGTCAACGGATTTG-3'), GAPDH reverse (5'-GTGATGGCATGGACTGTGGT-3').

### Data presentation and statistics

Promoter/reporter transient transfections were repeated at least three times and results were expressed as mean ± SE. Statistical differences was determined using the Student’s *t*-Test. Statistical significance was determined at the *p* < 0.05 level. The EMSA and ChIP experiments were repeated at least three times and one representative result was shown for each set of experiments.

## Results

### Identification of NGX6 minimal promoter (-86/+100)

**Figure 1:**
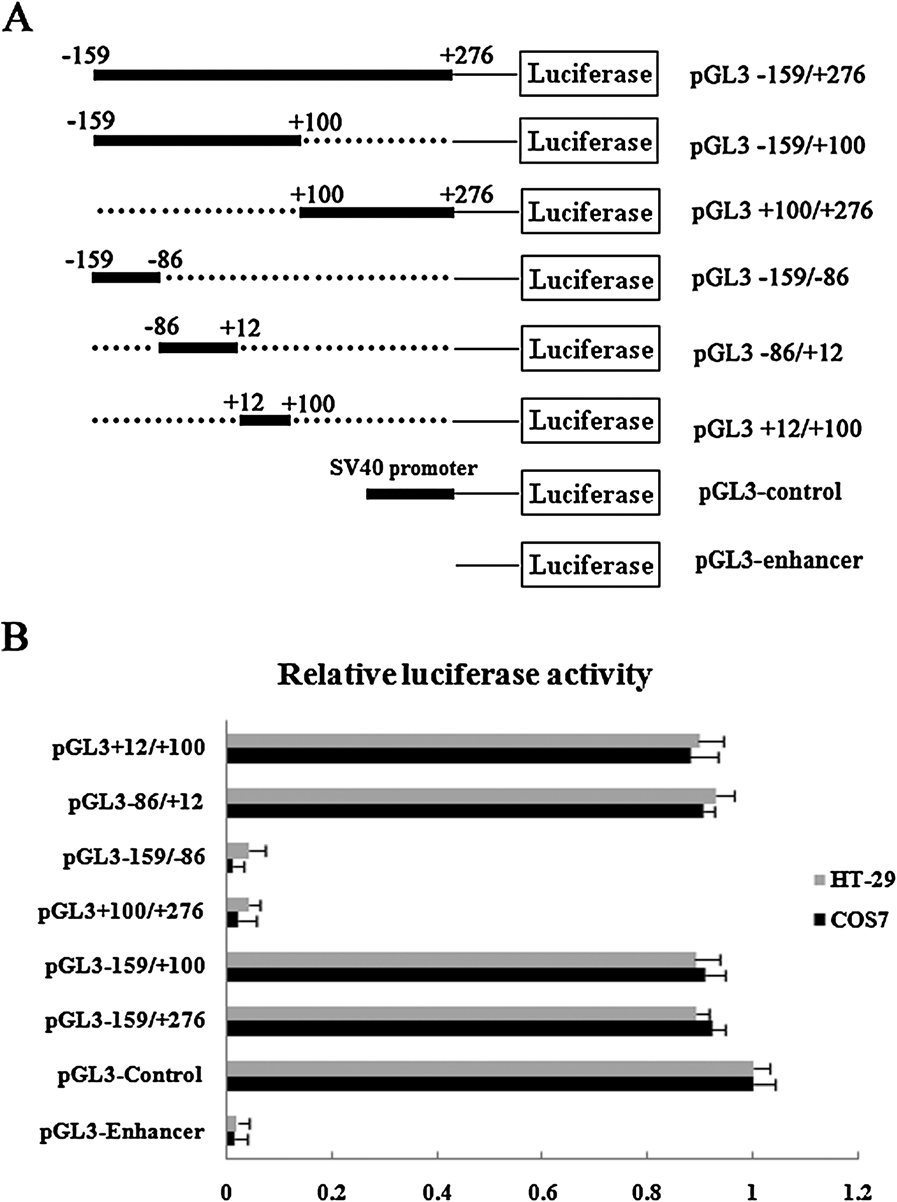
**Identification of NGX6 minimal promoter (-86/+100). (A)** Schematic illustration of deletion constructs of NGX6 proximal promoter pGL3-159/+276. **(B)** Luciferase activity of the deleted constructs in COS7 and HT-29 cells. pGL3-control, pGL3-enhancer and NGX6 promoter constructs were transfected into COS7 and HT-29 cells, respectively. All of the vectors were cotransfected with β-galactosidase vector for normalizing transfection efficiency. Luciferase activities were analyzed 48 hours post transfection. Data are expressed as mean ± SE of three independent experiments.

In order to define the minimal promoter of NGX6 gene, five progressive 5' ends deletion constructs were generated from the full-length promoter construct pGL3 -159/+276 (Figure 1A). Plasmid pGL3 -159/+276, pGL3 -159/+100, pGL3 + 100/+276, pGL3 -159/-86, pGL3 -86/+12 and pGL3 + 12/+100 were transiently transfected into COS7 and HT-29 cells, respectively. The luciferase activity driven by NGX6 promoter constructs was measured after transfection for 48 h. Luciferase expression levels were corrected for variable transfection efficiencies by cotransfection with β-galactosidase plasmid. As shown in Figure 1B, three constructs, pGL3 -159/+100, pGL3 -86/+12 and pGL3 + 12/+100 showed the same high luciferase expression as the full-length promoter construct pGL3 -159/+276, whereas luciferase expression in pGL3 + 100/+276 and pGL3 -159/-86 transfected cells was extremely low as pGL3-enhancer. These results indicated that a 186-bp fragment spanning positions -86 to +100 bp is required for the basal transcriptional activity of the NGX6 promoter.

### Transcription factor Sp1 and Egr-1 bind to an overlapping Sp1/Egr-1 binding motif in the NGX6 minimal promoter

**Figure 2:**
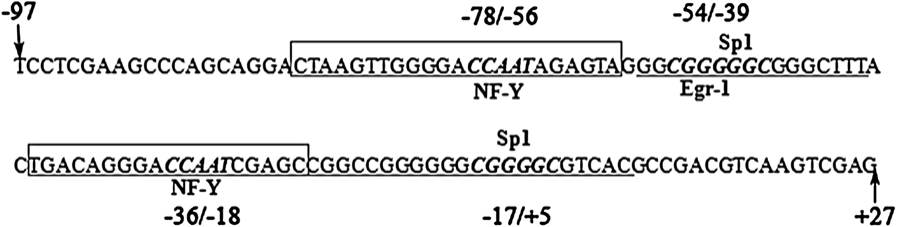
**Bioinformatics analysis of putative cis-acting elements in NGX6 minimal promoter region -86/+100 by MatInspector program.** A series of results were predicted, including Sp1 binding site(-17/+5), two NF-Y binding sites (-78/-56 and -36/-18) and a Sp1/Egr-1 overlapping site (-54/-39). The genomic sequence number 35819222 of NGX6 gene is defined as +1.

**Figure 3:**
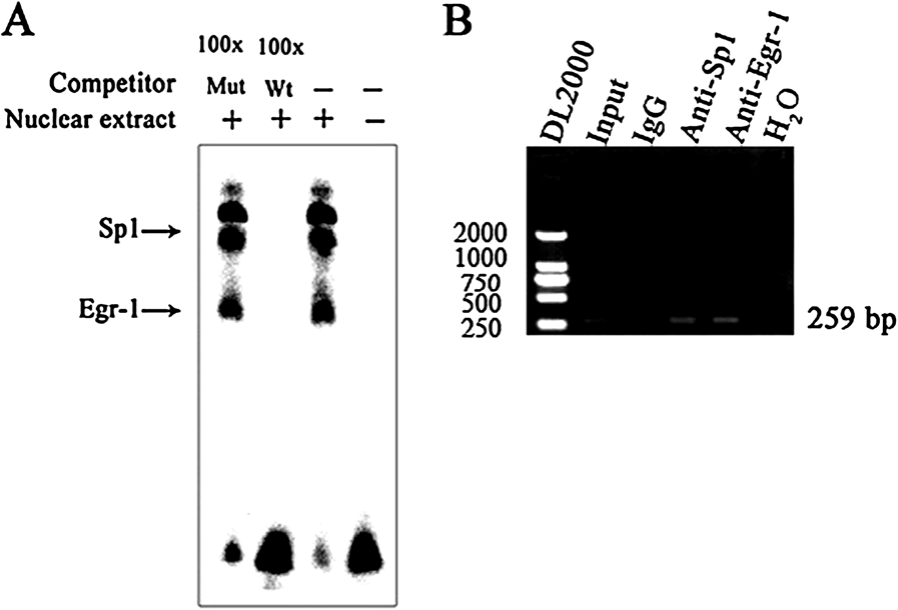
**Transcription factor Sp1 and Egr-1 bind specifically to the Sp1/Egr-1 overlapping site in NGX6 minimal promoter. (A)** Electrophoretic mobility shift assay demonstrating complex formation between the G/C-rich, potential Sp1/Egr-1 binding domain of NGX6 gene promoter in HT-29 cells. Oligonucleotides -54/-39 containing a Sp1/Egr-1 overlapping site was incubated with 10-μg nuclear extracts of HT-29 cells in EMSA. For competition assay, 5-pmol of unlabeled wild-type or mutated oligonucleotide was added to the reaction mixture, before the addition of labeled probes. **(B)** ChIP assay of the NGX6 promoter at the Sp1/Egr-1 overlapping binding site. Crosslinked chromation was sheared with nine 10-sec pulses at 10-sec intervals. Immunoprecipitation of chromatin fragments were carried out with anti-Sp1, and anti-Egr-1, respectively. Rabbit IgG was used as a negative control and Input was used as a positive control. After de-crosslinking, the purified DNA samples were analyzed by PCR. water (a reaction without any DNA) were used as a negative control for PCR.

A region spanning -86 to +100 bp of NGX6 minimal promoter was performed with MatInspector Professional program. Some critical putative binding sites were predicted in this region, including the binding site for Sp1 (-17/+5), two NF-Y binding sites (-78/-56 and -36/-18) and a Sp1/Egr-1 overlapping site (-54/-39) (Figure 2). To identify the binding of transcription factor(s) to this putative overlapping binding site within NGX6 promoter, EMSA and ChIP were performed. EMSA assay was conducted using a biotin 5' end-labeled oligonucleotide probe spanning -54 to -39 bp region. As shown in Figure 3A, three DNA protein complexes were detected after incubate the probe with nuclear extract from HT-29 cells. The complex formation was fully suppressed by the addition of a 100-fold molar excess of unlabeled wild type -54 to -39 bp oligonucleotide probe. This suppression was not observed when 100-fold molar excess of unlabeled mutated type -54 to -39 bp oligonucleotide probe was added as competitor. These results indicated that the Sp1/Egr-1 binding site at -54 to -39 bp is specific. To investigate whether transcription factor Sp1 and Egr-1 interacts with NGX6 minimal promoter through their specific binding sites in vivo, ChIP assay was performed. As shown in Figure 3B, with the DNA samples immunoprecipitated in HT-29 cells by rabbit polyclonal antibodies against Sp1 and Egr-1 respectively as templates, a 259 bp DNA fragment of NGX6 promoter could be amplified. These results further showed that transcription factor Sp1 and Egr-1 specifically bind to NGX6 minimal promoter.

### Role of the transcription factor Egr-1 in the regulation of NGX6 promoter activity

**Figure 4:**
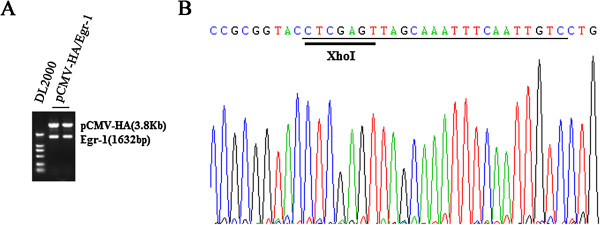
**Identification of pCMV-HA/Egr-1 vector. (A)** verfication of pCMV-HA/Egr-1 by enzyme cutting. Lane 1: DL 2000 molecular mass marker; lane 2 and 3: enzyme cutting of pCMV-HA/Egr-1 with EcoRI and XhoI. **(B)** The results of sequencing of pCMV-HA/Egr-1. The reverse primer was presented.

**Figure 5:**
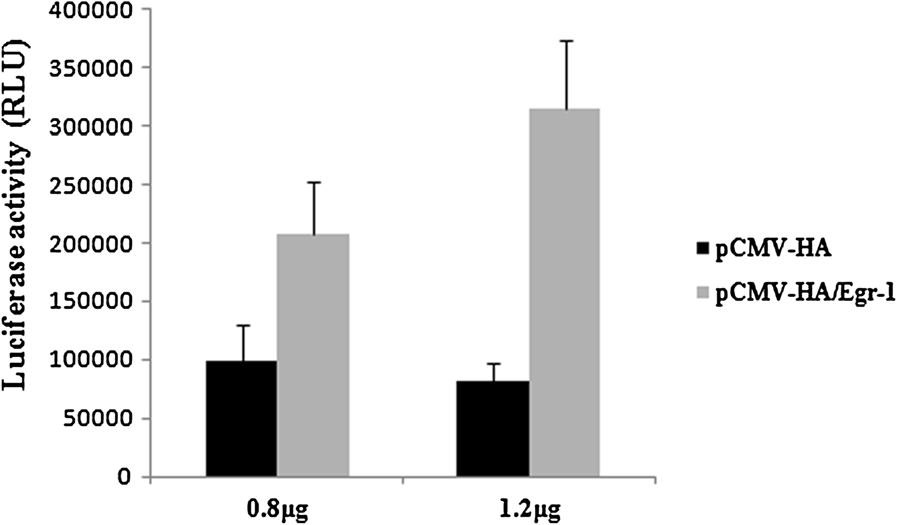
**Transcriptional factor Egr-1 increases NGX6 promoter activity in SW620 cells.** 0.5 μg pGL3-86/+12 vector was cotransfected with 0.5 μg SV40 β-galactosidase vector and various amount pCMV-HA/Egr-1 into SW620 cells by Lipofectamine 2000 Reagent. Luciferase activity was measured in cell extracts after transfection for 48 h. Then β-galactosidase was used for for normalizing transfection efficiency. Data are expressed as mean ± SE of three independent experiments.

In order to detect the effects of transcriptional factor Egr-1 on the promoter activity of NGX6 gene, we constructed plasmid pCMV-HA/Egr-1. The plasmid was confirmed by enzyme cutting (Figure 4A) and sequencing (Figure 4B). SW620 cells were transfected with 0.5 μg NGX6 promoter constructs pGL3 -86/+12, 0.5 μg SV40 β-galactosidase vector and various amount of pCMV-HA/Egr-1. As shown in Figure 5, overexpression of Egr-1 increased the promoter activity of pGL3-86/+12 in SW620 cells. Similar results were observed in HT-29 and SW480 cells (data no shown). These results indicated that transcription factor Egr-1 is a positive regulatory element for NGX6 gene.

### Requirement of Egr-1 for induction of NGX6 expression

**Figure 6:**
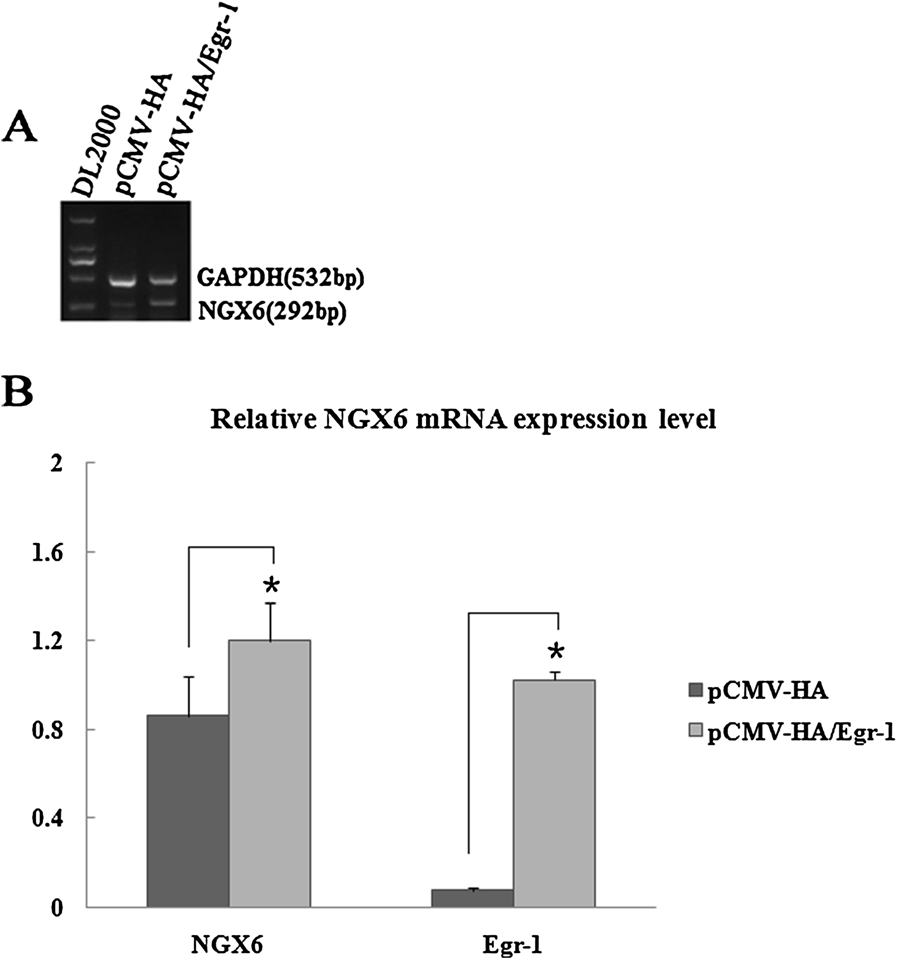
**Overexpression of Egr-1 down-regulated the mRNA Expression level of NGX6 gene in SW620 cells. (A)** mRNA expression of NGX6 was assayed by reverse transcription ploymerase chain reaction (RT-PCR). **(B)** mRNA expression level of NGX6 gene and Egr-1 were examined by Real-Time quantitative PCR. Results are presented as mean ± SE (n = 3). pCMV-HA was used as negative control. Statistically significant differnces from control levels are indicated by *(P < 0.05).

**Figure 7:**
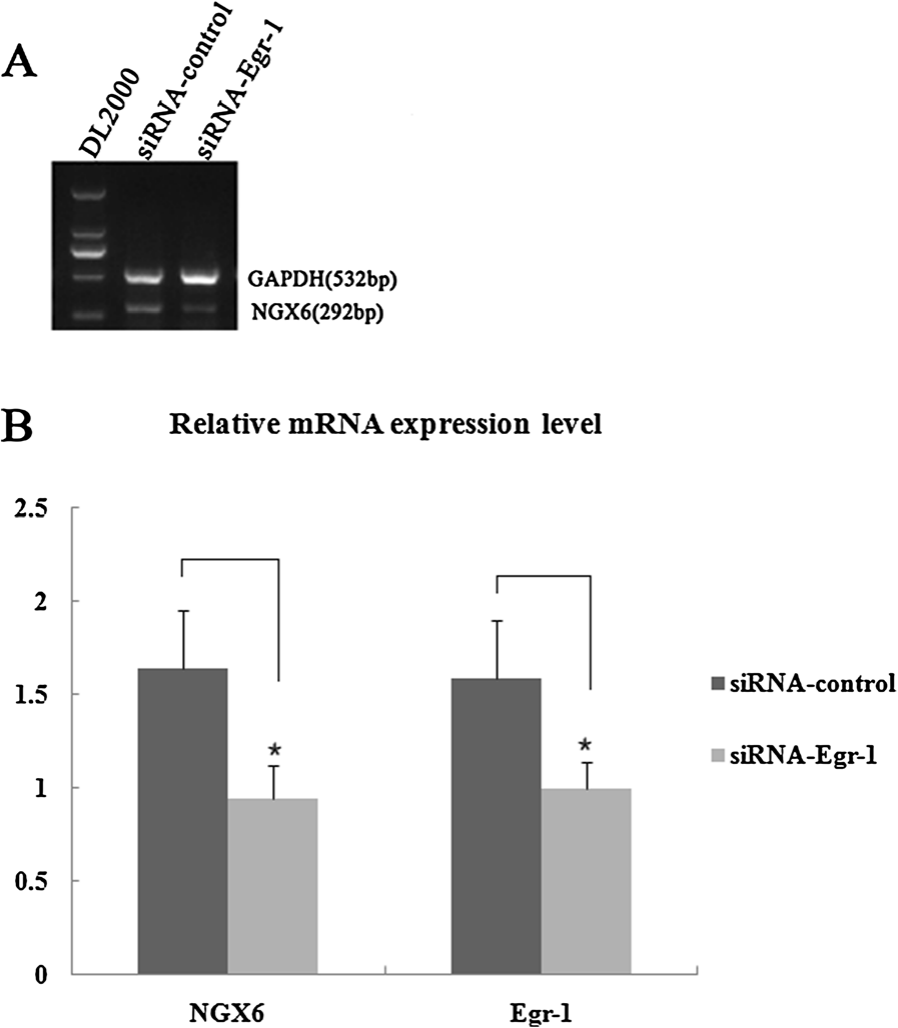
**Knockdown of endogenous Egr-1 decreased the mRNA Expression level of NGX6 gene in SW480 cells. (A)** Detection of NGX6 mRNA expression by RT-PCR. And the PCR products were analyzed by electrophoresis on a 5% agarose gal. **(B)** mRNA expression level of NGX6 and Egr-1 were examined by Real-Time quantitative PCR. Results are presented as mean ± SE (n = 3). siRNA-control was used as negative control. Statistically significant differnces from control levels are indicated by *(P < 0.05).

To investigate whether Egr-1 was required for induction of NGX6 gene expression, pCMV-HA/Egr-1 was transiently transfected into SW620 cells, and pCMV-HA was used as a negative control. 48 h after transfection, mRNA was isolated from the cells. NGX6 mRNA expression was detected by RT-PCR (Figure 6A) and Real-time quantitative PCR (Figure 6B). These results indicated that compared to overexpression of the control vector pCMA-HA, NGX6 mRNA level was increased when Egr-1 was overexpressed in SW620 cells.To investigate the role of endogenous Egr-1 in regulation of NGX6 expression, siRNA technology which small DNA inserts encoding short hairpin RNA against Egr-1 expression was used. SW480 cells were transfected with siRNA-Egr-1 or siRNA-control, respectively. 48 h after transfection, mRNA was isolated from harvested cells. RT-PCR (Figure 7A) and Real-time quantitative PCR (Figure 7B) revealed that compared with siRNA-control, transfection with siRNA-Egr-1 reduced the expression levels NGX6 mRNA. Taken together, these results indicated that Egr-1 is required for NGX6 mRNA expression.

## Discussion

NGX6 (genebank accession number AF188239), a novel candidate metastasis suppressor gene, was cloned by a location candidate cloning strategy. Its function is associated with colorectal carcinoma occurrence and development. Previous study has indicated that NGX6 was decreased or undetectable in colorectal carcinoma and involved in cellular growth, cell cycle progression and tumor angiogenesis. However, little is known about the transcriptional regulation of NGX6 gene.

Bioinformatics analysis indicated that no canonical TATA boxes were found in NGX6 promoter, while two CAAT boxes, a GpG island and putative transcription binding sites for Sp1, Egr-1, NF-Y etc. were discovered [13]. In this report, using various promoter deletion constructs, the minimal promoter of NGX6 gene was defined in a -86 to +100 bp region, which is the shortest promoter identified so far in the regulatory region of NGX6 gene. The results of MatInspector showed that this region contained an overlapping Sp1/Egr-1 GC-rich motif for the binding of the zinc finger transcription factors Egr-1 and Sp1. EMSA along with ChIP assays confirmed a specific Sp1/Egr-1 overlapping site spanning from -54 to -39 bp in NGX6 minimal promoter. Similar Sp1/Egr-1 overlapping binding sites have been shown to play a critical role in the expression of some genes, such as *TF*, *NDRG1* and *PDGF-A*[14-16]. The regulation of transcription by these two transcription factors has been shown to be complex: in some genes the two factors are synergistic, whereas in other systems the factors appear to compete [17,18]. Egr-1 encodes a nuclear phosphoprotein that binds to the GC-rich sequence 5'-GCGGGGGCG-3' and regulates transcription of target gene through the GC-rich consensus sequence [19]. Egr-1 expression had been found to be either decreased or undetectable in nasopharyngeal carcinoma and colorectal carcinoma [20,21]. Various studies have indicated Egr-1 is involved in regulation of cell proliferation and may have tumor suppressive functions [22,23]. In our experiment, immunohistochemical staining of Egr-1 showed weaker staining in metastatic tissue in comparison to non-metastatic tissue in a colorectal tissue microarray.

RT-PCR and Western Blot also confirmed that Egr-1 expression level in SW620 cells is lower than that in SW480 cells (data not shown). Therefore, we hypothesize that Egr-1 regulate the expression of NGX6 gene in colorectal cancer as a tumor suppressor gene. In our study, overexpression of Egr-1 increased the activity of NGX6 promoter and up-regulated the expression level of NGX6 mRNA, whereas knock-down of Egr-1 reduced endogenous mRNA expression of NGX6 gene in SW480 cells. From these findings, we conclude that Egr-1 is indeed a positive regulator of NGX6 gene. In previous study, a series of related experiment has revealed that nuclear transcription factor Sp1 also positively regulates NGX6 promoter transcriptional expression [13]. Egr-1 binding may influence the occupancy of Sp1 proteins in certain environment such as hypoxia [15] and result in the induction of NGX6 gene expression changes. Further studies needs to be done to confirm this hypothesis.

## Conclusions

In summary, the current study provides a molecular model for Egr-1 in positive regulation of NGX6 promoter activity and mRNA expression. These results will help to better understand the role of NGX6 gene in carcinoma progression and may provide a new potential therapeutic target for cancer therapy from the view of knockdown of Egr-1 and down-regulation of NGX6 gene.

## Competing interests

The authors declare that they have no competing interests.

## Authors’ contributions

MJL participated in the study design and coordination, data collection, drafting of the manuscript and carried out the bioinformatics analysis, EMSA and ChIP assays. YP helped the cell culture and the luciferase assay. gDNA extraction and DNA methylation analysis. XYW participated in experimental design, helped to draft the manuscript and carried out data interpretation. SRS and GYL carried out the experiment design, manuscript drafting and revision. All authors read and approved the final manuscript.
